# Observing the release of twist by magnetic reconnection in a solar filament eruption

**DOI:** 10.1038/ncomms11837

**Published:** 2016-06-16

**Authors:** Zhike Xue, Xiaoli Yan, Xin Cheng, Liheng Yang, Yingna Su, Bernhard Kliem, Jun Zhang, Zhong Liu, Yi Bi, Yongyuan Xiang, Kai Yang, Li Zhao

**Affiliations:** 1 Yunnan Observatories, Chinese Academy of Sciences, Kunming, Yunnan 650216, China; 2 Key Laboratory of Solar Activity, National Astronomical Observatories, Chinese Academy of Sciences, Beijing 100012, China; 3 School of Astronomy and Space Science, Nanjing University, Nanjing, Jiangsu 210093, China; 4 Key Laboratory for Dark Matter and Space Science, Purple Mountain Observatory, Chinese Academy of Sciences, Nanjing, Jiangsu 210008, China; 5 Institute of Physics and Astronomy, University of Potsdam, Potsdam 14476, Germany

## Abstract

Magnetic reconnection is a fundamental process of topology change and energy release, taking place in plasmas on the Sun, in space, in astrophysical objects and in the laboratory. However, observational evidence has been relatively rare and typically only partial. Here we present evidence of fast reconnection in a solar filament eruption using high-resolution H-alpha images from the New Vacuum Solar Telescope, supplemented by extreme ultraviolet observations. The reconnection is seen to occur between a set of ambient chromospheric fibrils and the filament itself. This allows for the relaxation of magnetic tension in the filament by an untwisting motion, demonstrating a flux rope structure. The topology change and untwisting are also found through nonlinear force-free field modelling of the active region in combination with magnetohydrodynamic simulation. These results demonstrate a new role for reconnection in solar eruptions: the release of magnetic twist.

## Abstract (web summary)

Magnetic reconnection is a fundamental process giving rise to topology change and energy release in plasmas, of particular relevance for the Sun. Here the authors report the observation of fast reconnection in a solar filament eruption, which occurs between a set of ambient fibrils and the filament itself.

## Graphical abstract

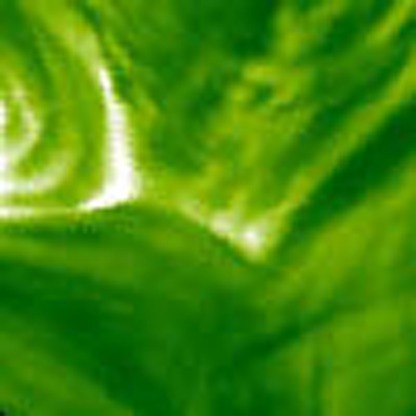

It is widely accepted that magnetic reconnection plays an important role in plasmas, particularly in solar eruptive events, such as flares^1^,^2^,^3^,^4^,^5^, filament eruptions^6^, coronal mass ejections^7^,^8^ and jets^9^. In the models of magnetic reconnection, magnetic field lines with an oppositely directed component approach each other in a current sheet or at a magnetic null point, break up and reconnect to form new magnetic lines. Magnetic energy is thereby released into thermal and kinetic energy of the plasma, potentially leading to large-scale phenomena. Evidence of reconnection on the Sun has mostly been limited to single aspects and has been indirect, showing changes of magnetic connections^10^, reconnection inflows^11^,^12^,^13^ and outflows^14^,^15^,^16^,^17^, hot cusp-shaped structures at their interface^11^,^13^,^14^, supra-arcade downflows^15^,^18^, loop shrinkage^19^,^20^, sudden brightenings^21^,^22^, current sheets^15^,^20^,^23^, plasmoid ejections^24^,^25^, loop-top hard X-ray sources^20^,^26^, pulsating radio emissions^27^, and coronal heating in the interface between emerging and ambient magnetic flux^28^.

In recent years, direct and more comprehensive evidence of reconnection has been discovered in a couple of energetic events on the Sun. Two very clear cases of reconnection, including inflowing cool loops, outflowing hot loops and plasma heated to >10 MK, were observed in solar flares through extreme ultraviolet (EUV) and X-ray data^12^,^13^. The reconnection inflow and outflow velocities could be inferred in these flares to lie in the ranges of 10–90 and 90–460 km s^−1^, respectively, constraining the reconnection rate to the range of 0.05–0.5. Inflows, outflows and the formation of new loops could also be studied in a well-observed case of reconnection between two sets of small-scale chromospheric loops, imaged at high resolution in H-alpha^22^. This study showed a transition from slow to fast reconnection. The three-dimensional (3D) topology of reconnecting loops and their heating has been inferred using the combined perspectives and multiple EUV channels of two spacecraft^29^.

Here we present comprehensive observational evidence of reconnection between a set of chromospheric fibrils and the threads of an erupting filament, which gave rise to a small flare. We observe the in- and outflows and hot cusp-shaped structures at the ends of a small-scale reconnecting current sheet, as well as newly formed loops that demonstrate the change of magnetic connectivity. We estimate that the reconnection is fast. The intriguing rotational motion of the erupted filament is found to show the untwisting of a flux rope, enabled by the reconnection with the chromospheric fibrils, which extend to essentially current-free magnetic flux in the corona. Our detailed study is made possible using H-alpha images from the New Vacuum Solar Telescope (NVST)^30^ and EUV images from the Solar Dynamics Observatory (SDO)^31^ (see the Methods section for details). Hot coronal plasma is also imaged using the X-Ray Telescope (XRT) on board Hinode^32^ and the Soft X-ray Imager (SXI) on board the Geostationary Operational Environmental Satellite (GOES)^33^. Photospheric vector magnetograms taken by the Helioseismic and Magnetic Imager (HMI)^34^ on board SDO allow us to obtain the 3D field structure of the reconnection region, independently demonstrating the change of topology. The dynamics of this source region model are studied in a data-constrained magnetohydrodynamic (MHD) simulation, which reproduces the observed features of the eruption and confirms that the untwisting of the erupted flux is enabled by reconnection with ambient current-free flux.

## Results

### Confined partial eruption of the filament

**Figure 1:**
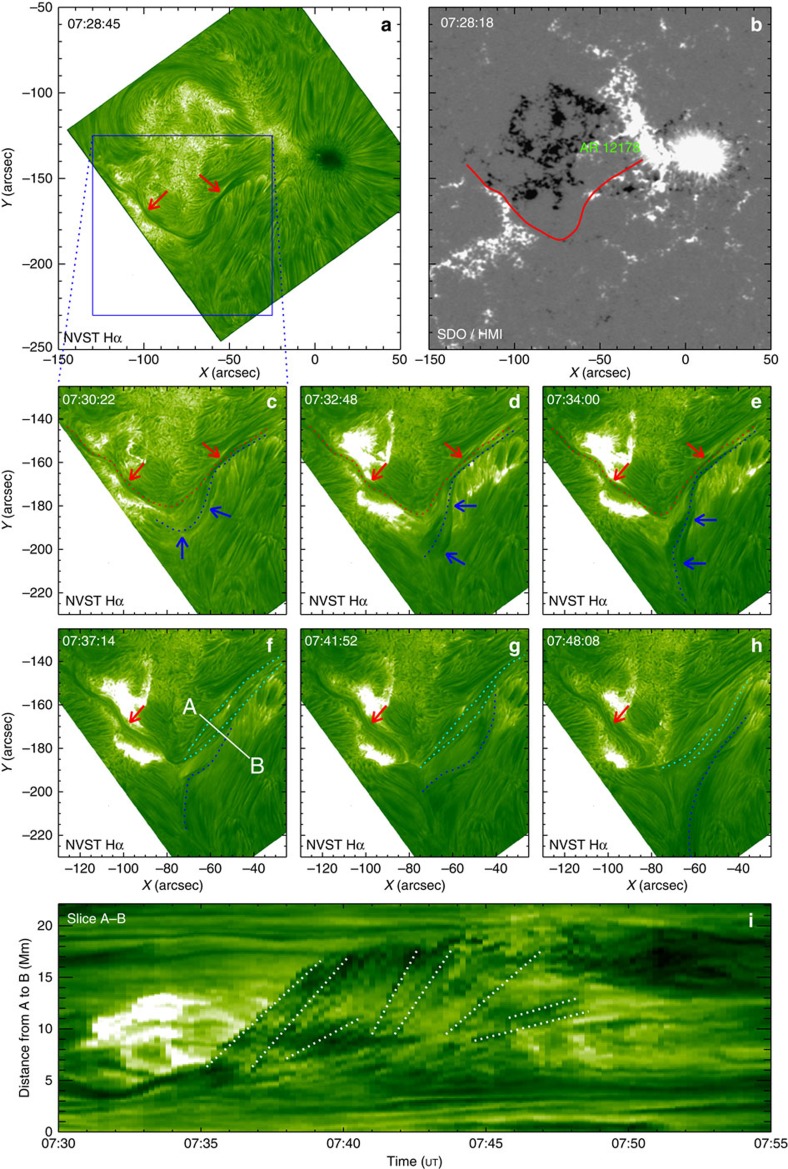
Structure and eruption of the filament. (**a**) NVST H-alpha image showing the general appearance of the filament (indicated by the red arrows) at the onset of the eruption. (**b**) HMI line-of-sight magnetogram of AR 12178, with positive (negative) photospheric flux shown in white (black) and the red line representing the position of the filament. (**c**–**h**) The process of filament eruption in H-alpha images. The original position is marked by the red-dotted line. The erupting part is indicated by the blue-dotted lines and blue arrows. Cyan-dotted lines in **f**–**h** indicate some of the filament threads that rise with a delay. The stable eastern filament section is marked by the red arrows in **f**–**h**. (**i**) Time-slice plot acquired at the position A–B, with the white-dotted lines marking the filament threads. The motion of these threads indicates the untwisting of the filament during its eruption.

On 3 October 2014, a filament is observed in active region (AR) 12178 (as designated by the National Oceanic and Atmospheric Administration [NOAA]) near the disk centre. The filament is composed of a western and an eastern section, which join smoothly at a bend point near solar (*x*,*y*)=(−70,−185). Many thin threads extending along the filament spine make up its fine structure (see the red arrows in Fig. 1a). The threads in the western section show indications of twist of approximately one turn, consistent with the widely but not universally adopted assumption that the magnetic structure of filaments is that of a weakly twisted magnetic flux rope^35^,^36^,^37^. Figure 1b shows the position of the filament in the magnetogram. Positive photospheric polarity is given at the western footpoint and southern side of the filament, and negative polarity is given at the eastern footpoint and northern side, so the filament has sinistral chirality^38^. This corresponds to positive (right handed) magnetic helicity, because the axial current of the filament must also point eastward for a force-free equilibrium to exist in the given ambient flux distribution.

Figure 1c–h and the related Supplementary Movie 1 show the eruption of the filament in H-alpha images taken by the NVST. The eruption commences near the bend point at ∼07:26 UT, as a motion of the dark threads with a southwestward projected direction, which later turns more southward. Associated brightenings, indicating heating due to reconnection, appear soon but only after the onset of the motion. The eruption comprises the whole western filament section and part of the eastern section. Some of the threads in the western section follow the main eruption with a small delay. The threads in the eastern section's part adjacent to the bend point (−100≲*x*≲−70) connect smoothly to the threads in the western section and move jointly with them (see the blue-dotted lines in Fig. 1c,d). Subsequently, other threads become visible in their place; these, as well as all other threads in the eastern filament section, remain stable (see the red arrows in Fig. 1f–h).

We conclude that part of the magnetic flux in the filament experiences an instability, which causes the motion and subsequently triggers reconnection. The unstable flux comprises the whole western section and part of the eastern section, where unstable flux lies on top of the stable flux. The unstable flux extends into the eastern section at least up to approximately *x*=−100 (the apparent end region of the erupting threads), but possibly up to approximately *x*=−120 (the extension of the triggered brightenings); the stable flux extends from the bend point to the edge of the active region. Because the indicated twist of approximately one turn lies below the threshold of the helical kink instability^39^,^40^ and the erupted filament does not build up a clear helical shape as a whole (see the blue-dotted line in Fig. 1c–h), the torus instability^41^ is the primary candidate mechanism for the onset of eruption.

All southward motions end by 07:50 UT, and all material is subsequently seen to slide down towards the western end point of the filament in this confined eruption, which does not produce a coronal mass ejection. Obviously, the southward end region of the erupted filament threads is the highest part of the visible erupted structure. The data do not definitely reveal whether the erupted flux is still magnetically rooted in the eastern filament section or if it now connects to other negative flux in the photosphere. However, the erupted threads point approximately towards the neighbouring AR 12179 in the southeast, away from the direction of the eastern filament section, but similar to pre-existing interconnecting loops between the two ARs. This is suggestive of reconnection between the erupting filament and these loops. After ∼09:30 UT, a new filament forms along the original western filament section, initially separate from the surviving filament in the eastern section, but 2 h later beginning to join it.

The eruption gives rise to two signatures of magnetic reconnection. The two prominent brightenings that develop at the sides of the eastern filament section (Fig. 1c–h) represent an indirect signature, which agrees perfectly with the reconnection assumed to release the energy in the so-called standard flare model^1^,^2^,^3^,^4^,^5^. Ambient flux passing over the filament is lifted; subsequently, its legs approach each other and reconnect in a vertical current sheet that is known to form under the erupting flux, but not imaged in the present event. The released energy is channelled downward along the field lines, producing the chromospheric brightenings where the reconnecting ambient flux is rooted. This can likewise be seen under the western filament section (Fig. 1d,e), but it is much weaker there, in agreement with the much smaller amount of ambient flux in this area (Fig. 1b).

After ∼07:38 UT, a second, direct signature of reconnection is revealed. Chromospheric fibrils south of the bend point reconnect with the trailing threads of the erupting western filament section on the north side of the bend point. The observations of the reconnecting current sheet are analysed in detail in the following section, where we find that it also forms under the erupting flux. However, here it is the erupting flux that reconnects with ambient flux, a process not envisioned in the standard flare model.

In addition, the erupted filament displays an intriguing motion that is highly suggestive of a rotation about its main direction (Supplementary Movie 1). The motion is also seen in the Atmospheric Imaging Assembly (AIA)^42^ data (Supplementary Movie 2). At least one full turn is indicated. The motion of the better visible threads on the upper side is also shown in a time-slice plot (white-dotted lines in Fig. 1i). When looking along the filament toward the western footpoint, the rotation is clockwise. Erupted flux is generally assumed to have the structure of a flux rope^5^,^6^,^7^,^8^. The positive helicity inferred above implies that the twist of the rope is right handed. The clockwise rotation thus represents an untwisting that is equivalent to the relaxation of magnetic tension and supports the conjecture of a flux rope structure for the erupted filament.

Erupting flux ropes show an apparent untwisting simply as the result of their expansion. While the total number of field line turns is preserved (in the absence of reconnection), the twist per unit length decreases. The stretching can appear as a propagation of twist if only a part of the rope displays a twist pattern. Although the threads of the erupting filament do not display a pronounced twist pattern in images (Fig. 1c–h), the animation in Supplementary Movie 1 is consistent with this effect in the southward expanding end region of the threads. The stretching can also mimic a rotation in a time-slice plot, as in Figure 1i. However, it cannot explain two effects visible in the present event: many threads shift nearly completely to the other side of the rope (cyan lines in Fig. 1g,h) and their southern end points appear to rotate about the rope axis.

True untwisting results from the conversion of twist into writhe of the flux rope axis and from reconnection with less twisted flux. The first effect is negligible here because a clear helical shape does not develop (Fig. 1c–h). Following reconnection with less twisted flux, the twist tends to equilibrate along the new structure. Again, the total number of turns present after the reconnection is preserved, but the equilibration occurs as a result of a true propagation of twist from the more twisted to the less twisted part (as a torsional Alfvén wave packet) and involves a true rotation about the axis of the new structure. Hence, the observed rotational motion indicates that the erupted filament flux reconnects with ambient flux, which usually has no twist. The H-alpha observations reveal only a small part of that flux (the chromospheric fibrils south of the bend point of the filament channel), but a consistent whole picture is provided below by our models for the coronal field of the AR and by the evolution of one of these models in an MHD simulation.

### Imaging small-scale magnetic reconnection

**Figure 2:**
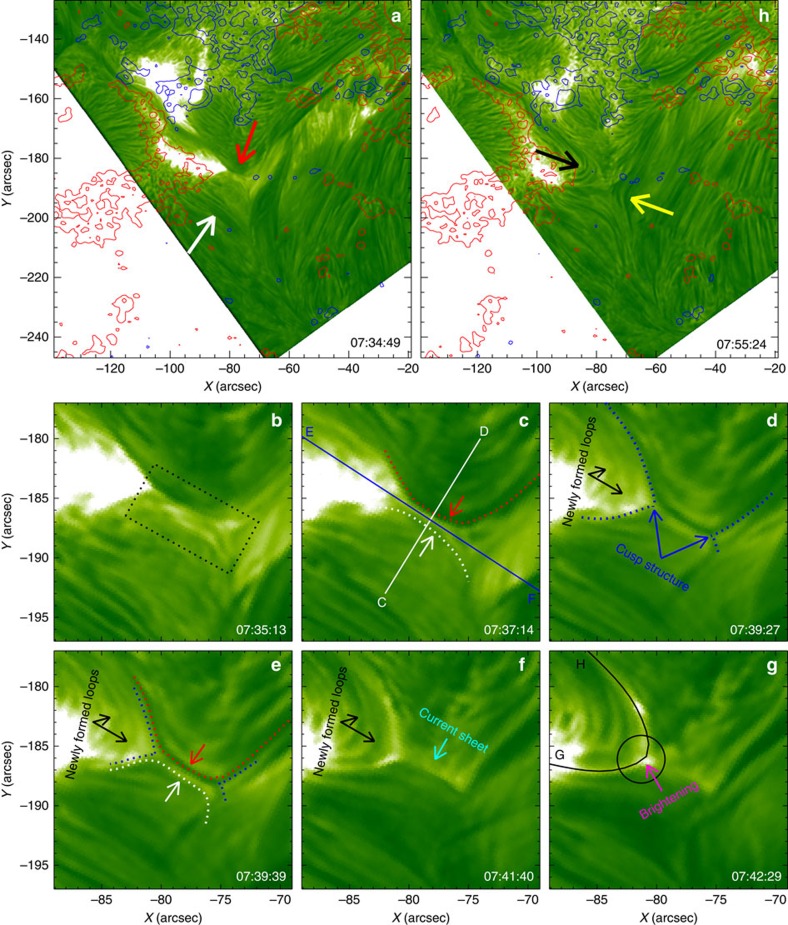
Evolution of reconnection. (**a**,**h**) NVST H-alpha images in the early and late phases of the reconnection event, overlaid by red (blue) contours representing positive (negative) photospheric polarity from near-simultaneous HMI data. (**b**–**g**) NVST H-alpha images of the reconnection process in which the red- and white-dotted lines indicate the magnetic loops before the reconnection (with red arrows marking the filament threads and white arrows marking the chromospheric fibrils), the blue-dotted lines mark the cusp-shaped structures, and the black (yellow), cyan and pink arrows point to newly formed loops, current sheet and brightening, respectively. The three time-slice plots shown in Fig. 4 are made along the white solid line C–D, the blue solid line E–F and the black solid line G–H, respectively.

The reconnection process and formation of the associated current sheet are shown in Fig. 2. Their full evolution can be better seen in Supplementary Movie 1. Figure 2a shows the positions of the original structures in an H-alpha image overlaid by a line-of-sight magnetogram. The reconnection occurs between two sets of magnetic loops with opposite directions (see the arrows in Fig. 2a). One set of magnetic loops, that is, the filament threads whose eruption is delayed, is indicated by the red arrow before the reconnection. The other set, that is, the chromospheric fibrils, is indicated by the white arrow. The reconnection occurs in a small region (marked by the black rectangle in Fig. 2b). In Fig. 2c, two typical loops (red- and white-dotted lines with red and white arrows, respectively) are indicated just before their reconnection. They further approach each other, and their interaction results in a reconnection at ∼ 07:38 UT. During the reconnection process, two cusp-shaped structures are formed (blue-dotted lines in Fig. 2d). Simultaneously, new loops appear on the other side of the cusp structure northeast of the reconnection region (the black arrows in Fig. 2d,e). Subsequently, these loops move away from the cusp. With the two sets of outer loops continuing to move towards each other, the reconnection continues from ∼07:38 to ∼07:50 UT. After the reconnection ceases, the newly formed loops can be seen more obviously (marked by black arrows in Fig. 2d–h). They have accumulated in the northeast reconnection outflow region. The new loops, which form early in the southwest of the reconnection region (at the right cusp), follow the filament eruption (Supplementary Movie 1), while those that form there later do not rise further and can be clearly seen near the end of the event (indicated by the yellow arrow in Fig. 2h).

**Figure 3:**
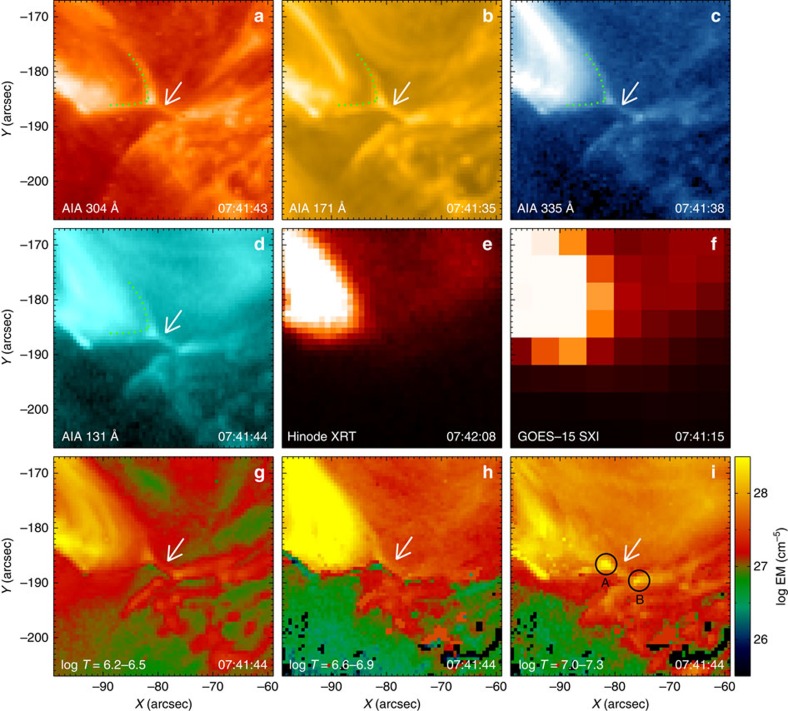
EUV/X-ray images and EM maps. (**a**–**d**) AIA EUV images at 304, 171, 335 and 131 Å channels, in which the cusp-shaped structures are marked by the dotted lines and the current sheet is indicated by the white arrows. (**e**,**f**) Cusp structure seen in X-ray images observed by Hinode/XRT and GOES/SXI, respectively. (**g**–**i**) EM maps in three temperature ranges showing the dominant temperatures of the inflow region, current sheet, cusps (encircled and marked ‘A' and ‘B' in **i**), and reconnected loops.

During the eruptive process the reconnection region is stretched, apparently to form a current sheet, which is visible in H-alpha as a bright linear structure extending between the tips of the two cusps (Fig. 2f). The average length and width of the current sheet are estimated to be 4.3 × 10^3^ and 1.06 × 10^3^ km, respectively. At the same time, we find that the plasma at the tip of the northeast cusp structure is significantly heated, causing the brightening (Fig. 2g). The cusp and current sheet can be seen in multiple channels (Fig. 3a–d; Supplementary Movie 2). The northeastern bright cusp appears very clearly in all EUV channels of AIA (*T*≈0.02−10 MK). The southwestern cusp structure is weaker, as is to be expected from lower plasma densities at the upper end of a current sheet that is formed and stretched out upward by the eruption. This also shows up only intermittently owing to absorption by the moving threads of the erupted filament, which demonstrates that the current sheet forms under the erupting flux. The current sheet appears as a bright linear structure in all AIA channels, most clearly at the highest temperatures (131 Å; *T*=11 MK). The hot cusp structure can also be clearly seen in the XRT and SXI images during the reconnection process (Fig. 3e,f). The unusual simultaneous visibility of the current sheet and cusp in H-alpha and EUV emissions (Figs 2f and 3a–d) is probably due to the fact that cool and dense threads and fibrils are embedded in the reconnection inflow. The major brightening of the current sheet and cusp in H-alpha immediately follows the inflow and fading of a major filament thread (prominent in the current sheet in Fig. 2c–e), suggesting heating of the thread by the reconnection.

The emission measure (EM) maps at the different temperature bins (Fig. 3g–i) show that the northeastern, newly formed loops are dominated by EM at temperatures of 4–8 MK, several times higher than the unperturbed corona. On the other hand, the tips of the two cusp structures (circles A and B) and the current sheet between them are most prominent at even higher temperatures, that is, *T*>10 MK (Supplementary Movie 3). This is similar to earlier observations of a pair of hard X-ray sources located at the two ends of the conjectured current sheet in flares^20^. The EUV images at high temperatures (Fig. 3d) and the EM maps (Fig. 3i) also show a diffuse area of enhanced temperature in the northwest inflow region after ∼07:35 UT, which begins to cool down ∼10 min later and then transiently brightens in H-alpha (Fig. 1h). The magnetic field inhibits heat conduction from the current sheet into the inflow region. Guided by the MHD simulation, which shows the formation of additional weak current layers north of the erupting filament, we interpret these enhancements as a signature of additional energy release in the complex ambient coronal field when it is perturbed by the eruption, followed by downward heat conduction.

**Figure 4:**
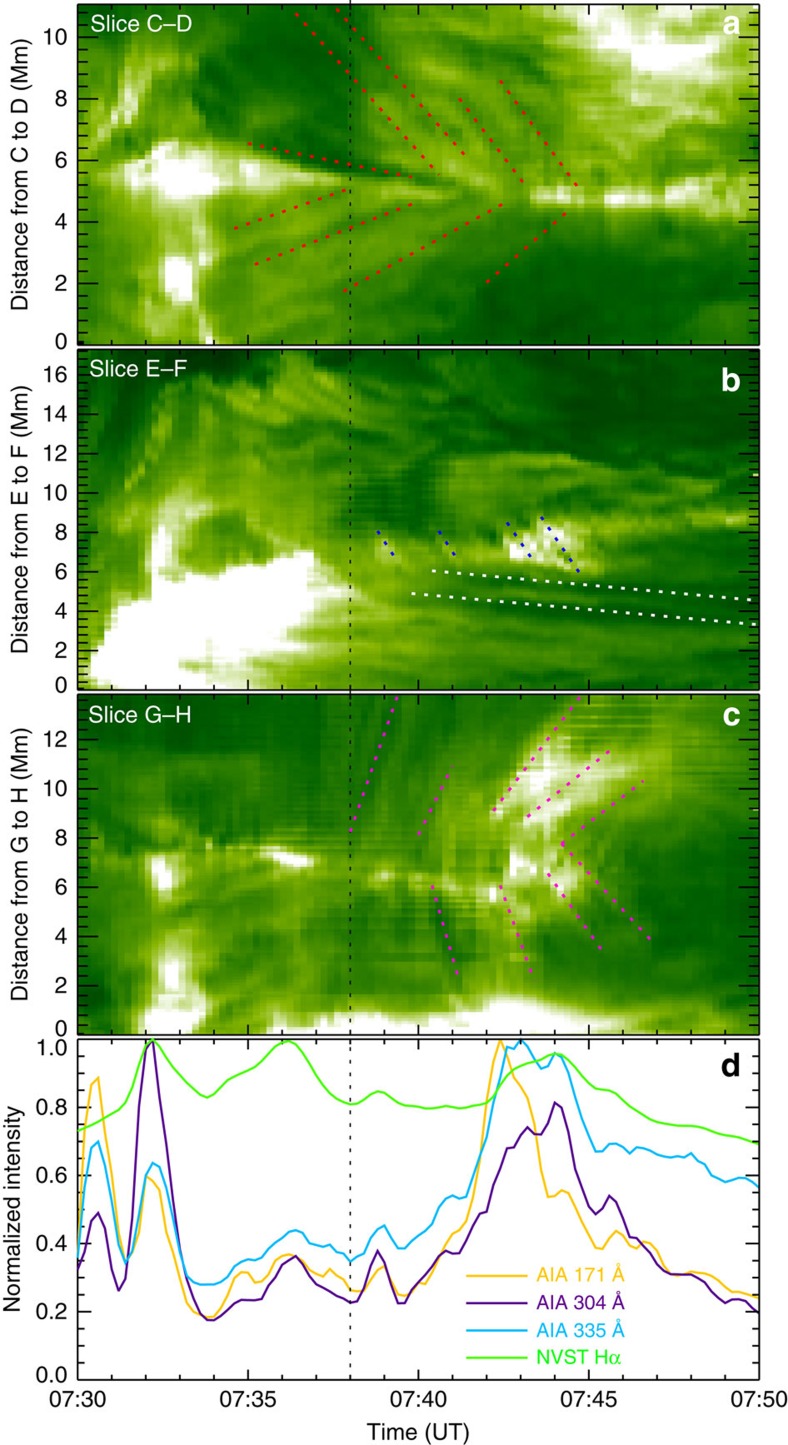
Time-slice plots and EUV brightening. (**a**–**c**) H-alpha time-slice plots made by stacking the slits along the slices C–D, E–F and G–H in Fig. 2, respectively. The red-, blue-, white- and pink-dotted lines represent reconnection inflows, outflows, shrinking loops, and downward motions, respectively. (**d**) Temporal evolution of the brightenings in the region marked by the black circle in Fig. 2g, using H-alpha, 304, 171 and 335 Å images, where each light curve is normalized by its maximum. The vertical black-dotted line in each panel indicates the onset of the observed fast reconnection.

To quantitatively investigate the motions of the plasma in the reconnection region, we construct the three time-slice plots in Fig. 4a–c, which yield projected velocities along the lines marked in Fig. 2. Figure 4a shows the inward motions of loops, that is, the reconnection inflow. The apparent velocities of these loops are in the range of 3.7–25.0 km s^−1^ for the various filament threads on the northwest side and 6.3–16.8 km s^−1^ for the various fibrils on the southeast side. Figure 4b displays outward motions of bright (hot) plasma, that is, the reconnection outflow, seen immediately downstream of the northeastern cusp. The outflow velocities lie in the range of 41.7–43.7 km s^−1^, with an average value of 42.7 km s^−1^. At the same time, two newly formed loops containing cooled plasma, dark in H-alpha (the white-dotted lines), can be seen in Fig. 4b. These shrink slowly, with an average velocity of 2.6 km s^−1^. In addition, a mixture of bright and dark plasma moves down the legs of the cusp structure, that is, in the interface between the reconnection inflow and outflow (Fig. 4c). This apparent mix of inflowing and outflowing plasma, as well as the intermittently irregular structure of the cusp legs (Supplementary Movie 1), whose observation is made possible by the high resolution of the NVST, provide support for the recent numerical finding of turbulence in reconnection developing at the separatrices^43^. The downflows at the cusps show velocities in the range of 17.6–80.2 km s^−1^ with a decreasing trend.

The brightenings at the end of the current sheet were observed in multiple channels. To investigate their evolution, Fig. 4d displays the intensities in the H-alpha, 304, 171 and 335 Å channels, integrated over the area of the circle in Fig. 2g. The early brightenings are caused by the eruption of the filament. The onset of reconnection, as marked by the rise of the EUV emissions at 07:38 UT, coincides with the arrival of the first inflow trace at the forming current sheet (Fig. 4a) and with the onset of outflows from the current sheet (Fig. 4b,c). The bright structures and the outflows show some degree of intermittency, which is a characteristic of plasmoid-mediated reconnection in very long current sheets, but here is probably caused mainly by the inhomogeneity of the inflowing plasma. All tracers of the reconnection process decay after ∼07:45 UT, by which time the length of the current sheet has shortened considerably because of the accumulation of new loops in the downward (northeast) outflow region and the approximate stationarity of the upper tip of the sheet under the erupted but no longer rising filament.

### 3D NLFFF configuration of the reconnection region

**Figure 5:**
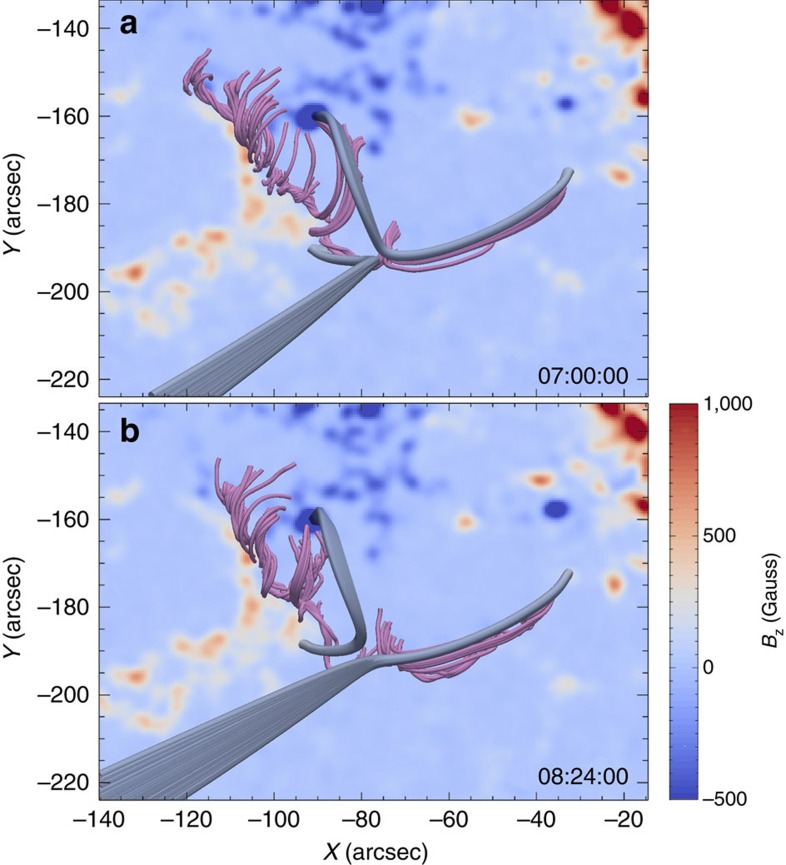
3D configuration of the reconnection region from NLFFF extrapolation. (**a**,**b**) 3D NLFFF configuration before (07:00 UT on 3 October 2014) and after (08:24 UT on 3 October 2014) the reconnection. The background images indicate the radial component of the vector magnetic field in the photosphere. The grey lines represent the magnetic loops involved in the reconnection. The pink lines show the magnetic structures of the filament.

To investigate the 3D magnetic field structure of the reconnection region, we carry out extrapolations of the HMI vector magnetograms at 07:00 and 08:24 UT based on the nonlinear force-free field (NLFFF) assumption for the coronal field (Fig. 5). In the eastern section of the filament, an incoherent flux rope structure is obtained, similar to several recent extrapolation results for active regions before an eruption^44^,^45^. In the western section, the strong axial flux expected in a filament is found, but the poloidal (twist) field component is largely missing. We expect that the latter results from the low signal-to-noise ratio in the extended area of the weak field around the western filament section (Fig. 1b). The overall shape of the filament is nevertheless well matched. The extrapolation also correctly indicates that the field in the area of the western filament section has a similar structure sometime after the eruption, providing the prerequisite for the observed formation of a new filament in the western section. These findings yield confidence in the large-scale structure of the extrapolated NLFFF.

**Figure 6:**
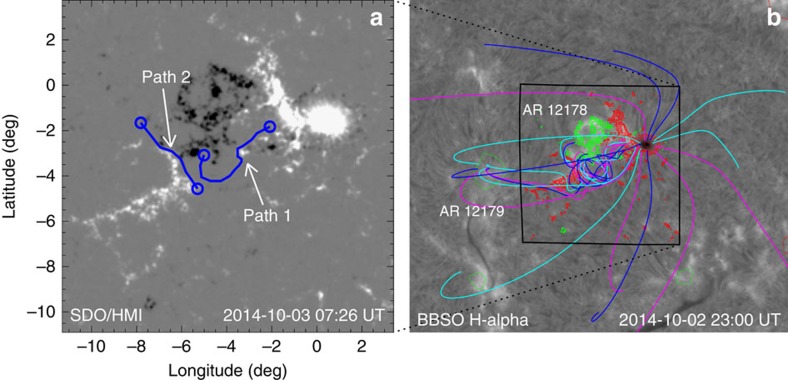
Magnetic field model constructed using the Flux Rope Insertion method. (**a**) Line-of-sight magnetogram observed by SDO/HMI at 07:26 UT on 3 October 2014. The two blue curves with a circle at each end show the paths of the inserted flux ropes. (**b**) H-alpha image taken by Big Bear Solar Observatory at 23:00 UT on 2 October 2014, overlaid with red (positive) and green (negative) contours representing the magnetic field shown in **a**. The colour lines show selected field lines from the magnetic field model, and the horizontal size of the MHD computation box (height of 0.75*R*_⊙_ and fully within the high-resolution region) is indicated by the black square.

The extrapolations demonstrate the change of magnetic connections in agreement with the observed change. They further show that the ambient field rooted in the southeast reconnection inflow region (grey field lines in Fig. 5a) first follows the low-lying reconnecting fibrils seen in H-alpha in the westward direction, but then bends strongly upward, forming high-reaching connections to the neighbouring AR 12179 (see Fig. 6). These field lines exchange footpoints, that is, reconnect, with a set of lower field lines running in the northwest inflow region and the western section of the filament channel (also coloured grey). The resulting low-lying loop (Fig. 5b) corresponds very well to the newly formed H-alpha loops downstream of the observed cusp (Fig. 2d–g). The other resulting set of field lines indicates that flux in the erupting western part of the filament reconnects with the high-reaching field lines that extend to the neighbouring active region. This corresponds very well to the southward bending of the erupting flux (blue-dotted lines in Fig. 1c–h) and supports our conjecture above that the strong untwisting motion is realized by a torsional Alfvén wave packet propagating to ambient, essentially untwisted flux.

### MHD simulation

**Figure 7:**
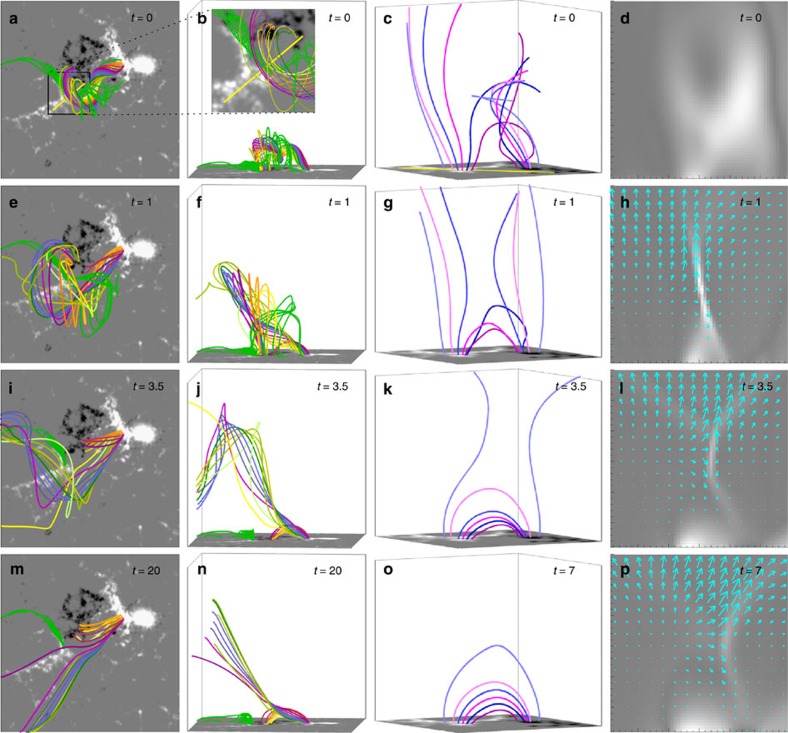
MHD simulation of the confined filament eruption and magnetic reconnection. The left two columns show field lines of the fully erupting western flux rope section (rainbow colours) and the largely stable eastern flux rope section (green) and the line-of-sight magnetogram in a cube of 0.25*R_⊙_* per side. The inset at *t*=0 indicates the area shown in the third column, and the yellow line shows the position of the vertical cut plane in the fourth column. The third column shows selected field lines in the area of the observed reconnection in a cube of 0.06*R*_⊙_ per side. Field lines rooted in positive photospheric flux (white) initially mostly extend to AR 12179 eastward of AR 12178 (Fig. 6b), while field lines rooted in negative flux (black) initially mostly join the unstable western flux rope section. The fourth column displays the current density and in-plane velocity vectors in a vertical cut through the reconnecting current sheet (0.06*R*_⊙_ per side). At *t*=0, the inserted, unstable flux rope is seen, with velocities set to zero. Times are given in Alfvén time, which corresponds to ∼3 min when scaled to the observations.

To further substantiate the occurrence of reconnection triggered by the erupting filament, we model the event in an MHD simulation whose initial and boundary conditions are constrained by the HMI data. Because the extrapolation largely fails to reproduce the twist in the western part of the filament, we construct a new NLFFF model of the AR from the pre-eruption magnetogram using the Flux Rope Insertion method, as detailed in the Methods section. On the basis of the observation that the western filament section erupts fully and the eastern section only partly, two flux ropes are inserted along the path of the filament. A low-lying rope is inserted along the eastern section. A higher-lying rope models the western section and extends into the eastern section as far as the moving threads at the onset of the event (Fig. 6a). Numerical relaxation partly merges them into a smooth configuration, which is essentially a flux rope split into two branches in the eastern part. From models for a range of values of the inserted fluxes, we select the one that yields an unstable western section, with approximately one field line turn, and a stable eastern section to serve as the initial condition of the MHD simulation (Figs 6b and 7a–d).

The model also includes the high-reaching field lines anchored in positive flux southeast of the filament (grey in Fig. 5a and left set of open field lines in Fig. 7c), the bottom part of which represents the reconnecting chromospheric fibrils (white arrows in Fig. 2a,c,e). Figure 6b shows that these field lines connect to the neighbouring AR 12179. Similarly, the low-lying field lines north of the filament bend point (grey in Fig. 5a) are here also seen to be part of the flux in the western filament section (right set of weakly twisted field lines in Fig. 7c). This flux models the trailing threads of the erupting western filament section (red arrows in Fig. 2a,c,e), which reconnect with the chromospheric fibrils.

A wide range of agreement with the observations is obtained. The simulation shows a confined eruption in the southern direction, comprising the full western and part of the eastern filament section (Fig. 7; Supplementary Movie 4). Magnetic reconnection is triggered very similarly to that shown by the NVST and SDO data. A current sheet is dragged upward into the corona between the rising and remaining flux along the filament section eastward of the original bend point (Fig. 7, fourth column). The ambient flux on the southeast side of the filament moves towards the sheet to reconnect at the observed position with approaching flux from the western section of the flux rope (Fig. 7, third column). As shown above, while being in agreement with the NVST observations, this differs partly from the standard view of reconnection in solar eruptions, which supposes that only ambient flux reconnects under a rising flux rope^5^,^6^,^7^,^8^,^46^. The reconnection forms small loops that accumulate under the current sheet at the position of the newly formed H-alpha loops (Figs 2d–g, 5b and 7o). The other parts of the reconnected field lines yield new, high-reaching magnetic connections from the western footpoint of the erupted flux to AR 12179 (Fig. 7, left two columns; compare with the evolution of the blue-dotted line in Fig. 1c–h). The untwisting of the erupted flux via propagation of twist along these new connections is very clear. These results are similar to the indications from the dynamics of the erupted filament in the H-alpha data (the draining of filament material in the westward direction along the whole length of the erupted filament and the indication of a rotational motion). They are also in agreement with the new connections in the extrapolated field (Fig. 5b). The accumulation of reconnected flux causes a gradual rise of the cusp that forms at the bottom tip of the current sheet (Fig. 7h,l,p). As the rise of the erupted flux is stalled, but the bottom cusp continues to rise as a result of the reconnection, the current sheet weakens and eventually decays.

The simulation shows further reconnection in a current sheet forming in the western filament section in the interface between rising and overlying flux. This rebuilds connections from the positive footpoint area of the flux rope to the strong negative photospheric flux patches adjacent to the eastern filament section (Fig. 7i,m). The current sheet is not imaged by NVST and SDO because it is not aligned with the line of sight; however, the resulting connections match the filament that reforms in the western section after 09:30 UT.

## Discussion

In this paper, we study in detail a small-scale magnetic reconnection event triggered by a filament eruption and obtain a comprehensive set of solid observational evidence for the occurrence of reconnection which is unprecedented. This includes the reconnection inflows at both sides of the current sheet at projected velocities of 3.7–25.0 km s^−1^; the reconnection outflow at one side of the current sheet, moving downward at projected velocities of 41.7–43.7 km s^−1^; the formation of a long, thin structure suggestive of a current sheet and of two cusp-shaped structures at its end points, both occurring simultaneously with the onset of reconnection; newly formed loops in both outflow regions, demonstrating the change of magnetic connectivity, those on the downward side shrinking with an average projected velocity of 2.6 km s^−1^; and fast downward motion at projected velocities of 17.6–80.2 km s^−1^ of cool and hot plasma at the cusp-shaped boundary between inflow and outflow. Moreover, the newly formed loops, current sheet and cusps show enhanced emission measure in a broad temperature range, with the loops being dominated by plasma at 4–8 MK and the current sheet and cusps by plasma at *T*>10 MK. The length, *L*, and width, *d*, of the current sheet are estimated to be 4.3 × 10^3^ and 1.06 × 10^3^ km, respectively.

The reconnection rate is an important physical parameter in the theory of reconnection and can be estimated from our observations. It can be expressed as the Alfvénic Mach number, *M*_A_, of the inflow velocity because the reconnection outflow generally approaches the Alfvén velocity, *V*_A_ (ref. 47). Assuming that the observed outflow velocity is of the order of *V*_A_ and neglecting projection effects, *M*_A_≈*V*_in_/*V*_out_, which lies in the range of ∼0.08−0.60 for our measurements. In a steady-state reconnection, the reconnection rate also equals the inverse aspect ratio of the current sheet^47^, which is obtained as *d*/*L*≈0.25, consistent with the estimated range for *M*_A_. Both indicate that the reconnection is fast. Our estimate tends to be relatively high, but is consistent with the range of previous estimates for solar events, for example, 0.001–0.03 (ref. 11), 0.01–0.23 (ref. 48), 0.055–0.20 (ref. 12), 0.16 (ref. 17) and 0.05–0.5 (ref. 13). Because the measured reconnection outflow in our event hits the accumulating newly formed loops after only a short distance (Fig. 2), it may not have reached the Alfvén velocity. Furthermore, the current sheet width obtained from the images, although resolved by the NVST, is considered an upper limit for the actual width of the field reversal, which tends to occur at microscopic scales^47^. Therefore, the estimated reconnection rate is also considered an upper limit.

Finally, the confinement of the eruption may be due to a strong restraining force of the overlying flux or due to the cancelation of the upward Lorentz force in the filament by its reconnection with ambient flux. The former effect is equivalent to saturation of the torus instability in an ambient field that decreases too slowly with height, which can be quantified by the decay index, *n*(*z*)=−*d* log* B*_p, hor_(*z*)/*d *log *z*, where *B*_p_,_ hor_(*z*) is the horizontal component of the potential field at the *x*–*y* position of interest^41^. The decay index profile at the observed position of the current sheet indicates instability (*n*>3/2) in a small interval around *z*=0.03*R*_⊙_, where the upper flux rope is located in our NLFFF model, and at *z*>0.13*R*_⊙_, with the intermediate height range being stable. In this stable range, the flux rope reconnects in our MHD simulation. Thus, both potential reasons for confinement may be relevant in the event.

## Methods

### Differential emission measure calculation

We calculate the differential emission measure (DEM) using the almost simultaneous observations of six AIA EUV lines (131, 94, 335, 211, 193 and 171 Å) formed at coronal temperatures. The DEM is determined by


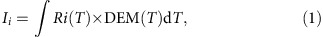


where *I*_*i*_ is the observed intensity of the waveband *i*, *R*_*i*_(*T*) represents the temperature response function of waveband *i*, and DEM(*T*) is the DEM of coronal plasma, which is computed using the routine xrt_dem_iterative2.pro in the Solar Software package. This code was first written for the Hinode/X-ray telescope data^49^,^50^, and then modified for the SDO/AIA data^51^. In this work, log *T* is set in the range of 5.5–7.5, where the DEM is generally well constrained^52^,^53^. To obtain the emission measure in the temperature range (*T*_min_,*T*_max_), we evaluate


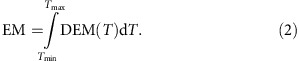


### NLFFF extrapolation

The 3D NLFFF structures of the reconnection region and associated filament (Fig. 5) are reconstructed using an optimization algorithm^54^,^55^. A preprocessing procedure is first used to deal with the bottom vector data to remove most of the net force and torque that usually results in an inconsistency between the photospheric magnetic field and the force-free assumption in the NLFFF models^56^. The visualization of 3D magnetic field is realized by the software Paraview. The field lines shown are traced from nearly identical points in the photosphere.

### Flux rope insertion method and MHD simulation

The magnetic field model used as the initial condition of the MHD simulation is constructed through Flux Rope Insertion. This method involves computing the potential field in the volume of interest by extrapolation, inserting a magnetic flux rope along the observed path of the filament and numerically relaxing the configuration to an NLFFF^36^,^57^,^58^.

The active region of interest (Fig. 6a) is modelled with high spatial resolution (0.002*R*_⊙_), and the more distant regions are modelled with a lower-resolution (1°) global potential field. The high-resolution region is derived from the line-of-sight photospheric magnetogram taken by SDO/HMI on 3 October 2014 at 07:26 UT, and the global field is constructed based on the HMI synoptic map. The high-resolution computational domain extends about 25° in longitude, 15° in latitude and up to 1.75 *R*_⊙_ from Sun centre. Two flux ropes are inserted along the path of the filament (blue lines) and anchored in the photosphere (at the blue circles), with path 1 chosen to run higher than path 2. The axial and poloidal fluxes along paths 1 and 2 are 4 × 10^20^ Mx and 10^11^ Mx cm^−1^, and 10^20^ Mx and 10^10^ Mx cm^−1^, respectively, giving the western section higher free magnetic energy and twist. The numerical relaxation partly merges the flux ropes into a smooth configuration and changes the end positions slightly (Fig. 6b). All flux connecting to photospheric sources outside AR 12178 corresponds well to the large-scale coronal loops imaged in the AIA 171-Å channel (which are not shown here, but can be accessed at the public source http://helioviewer.org/).

The MHD simulation code^59^ is used with numerical settings analogous to an earlier data-constrained simulation of a solar eruption^60^. In particular, the velocity in the bottom plane is set to zero, to model the inertia of the photosphere. The vertical component of the HMI magnetogram thus remains invariant.

### Software availability

The Solar Software package is available at http://www.lmsal.com/solarsoft/.

### Data availability

The NVST is a ground-based telescope with a 986-mm clear aperture in the Fuxian Solar Observatory of the Yunnan Observatories, Chinese Academy of Sciences. It is designed to observe the fine structures on the Sun and their activities in multiple layers (photosphere and chromosphere) with high spatial resolution (0.165 arcsec per pixel) and high temporal resolution (∼12 s). We use data from the H-alpha line-centre channel at 6,562.8 Å, corresponding to chromospheric temperatures. These data can be accessed at http://fso.ynao.ac.cn/dataarchive_ql.aspx. EUV and far-ultraviolet images obtained by the AIA on board SDO, including the 304, 171, 193, 335, 211, 94, 131 and 1,600 Å channels, display magnetic reconnection and the associated current sheet at higher temperature, that is, primarily in the corona. Photospheric magnetograms are provided by the HMI Instrument on SDO. The SDO data are publicly available at http://jsoc.stanford.edu/ajax/lookdata.html and at http://helioviewer.org/. Soft X-ray images of the Sun by Hinode/XRT and GOES/SXI are available at http://darts.isas.jaxa.jp/solar/hinode/query.php?A01=Go%20to%20Search/ and http://sxi.ngdc.noaa.gov/sxi/servlet/sxisearch/, respectively.

## Additional information

**How to cite this article:** Xue, Z. *et al.* Observing the release of twist by magnetic reconnection in a solar filament eruption. *Nat. Commun.* 7:11837 doi: 10.1038/ncomms11837 (2016).

## Supplementary Material

Supplementary Movie 1 The NVST H-alpha movie. (a) The general appearances of the filament and its eruption in which the red rectangle indicates the field of view (FOV) of panel b. (b) The process of magnetic reconnection.

Supplementary Movie 2 The EUV channel movie observed by SDO/AIA. (a-h) 304, 1600, 171, 193, 211, 335, 94, and 131 Å channel movies respectively.

Supplementary Movie 3 The EM map movie. In each panel, the EM maps at the different temperatures are shown.

Supplementary Movie 4 MHD simulation of the filament eruption. Field lines of the two flux ropes representing the unstable (rainbow-colored) and stable (green) filament sections are shown in vertical and perspective views (snapshots of which are shown in the left two columns in Fig. 6. The HMI line-of-sight magnetogram is included in the bottom plane.
